# RNA-binding protein RBM3 prevents NO-induced apoptosis in human neuroblastoma cells by modulating p38 signaling and miR-143

**DOI:** 10.1038/srep41738

**Published:** 2017-01-30

**Authors:** Hai-Jie Yang, Fei Ju, Xin-Xin Guo, Shuang-Ping Ma, Lei Wang, Bin-Feng Cheng, Rui-Juan Zhuang, Bin-Bin Zhang, Xiang Shi, Zhi-Wei Feng, Mian Wang

**Affiliations:** 1 School of Life Science and Technology, Xinxiang Medical University, Xinxiang 453003, China; 2 Henan Collaborative Innovation Center of Molecular Diagnosis and Laboratory Medicine, Xinxiang Medical University, Xinxiang 453003, China; 3 Henan Key Lab of Biological Psychiatry, Second Affiliated Hospital of Xinxiang Medical University, Xinxiang 453003, China

## Abstract

Nitric oxide (NO)-induced apoptosis in neurons is an important cause of neurodegenerative disease in humans. The cold-inducible protein RBM3 mediates the protective effects of cooling on apoptosis induced by various insults. However, whether RBM3 protects neural cells from NO-induced apoptosis is unclear. This study aimed to investigate the neuroprotective effect of RBM3 on NO-induced apoptosis in human SH-SY5Y neuroblastoma cells. Firstly, we demonstrated that mild hypothermia (32 °C) induces RBM3 expression and confers a potent neuroprotective effect on NO-induced apoptosis, which was substantially diminished when RBM3 was silenced by siRNA. Moreover, overexpression of RBM3 exhibited a strong protective effect against NO-induced apoptosis. Signaling pathway screening demonstrated that only p38 inhibition by RBM3 provided neuroprotective effect, although RBM3 overexpression could affect the activation of p38, JNK, ERK, and AKT signaling in response to NO stimuli. Notably, RBM3 overexpression also blocked the activation of p38 signaling induced by transforming growth factor-β1. Furthermore, both RBM3 overexpression and mild hypothermia abolished the induction of miR-143 by NO, which was shown to mediate the cytotoxicity of NO in a p38-dependent way. These findings suggest that RBM3 protects neuroblastoma cells from NO-induced apoptosis by suppressing p38 signaling, which mediates apoptosis through miR-143 induction.

Excessive generation of nitric oxide (NO) induces neural cell apoptosis, which can cause a broad range of neurodegenerative diseases^1^,^2^,^3^. NO is closely linked to mitochondrial damage, controlling the release of neurotransmitters and neuroendocrine secretion in neurodegenerative diseases, such as Parkinson’s disease, Alzheimer’s disease, and Huntington’s disease. Alteration of NO in the brain also interferes with key enzymes of tricarboxylic acid cycle and mitochondrial calcium metabolism, leading to an energy-deficient state and cell death in sequence^4^,^5^.

Sodium nitroprusside (SNP) serves as an NO donor and so induces apoptosis in neurons or neuroblastoma cells, to investigate the protective effect of various drugs^6^,^7^,^8^,^9^,^10^. The excessive NO derived from SNP induces neural cell apoptosis, which is involved in various signaling pathways, such as mitogen-activated protein kinases (MAPKs) p38, extracellular signal-regulated kinase (ERK), c-Jun N-terminal kinase (JNK), and AKT signaling and adenosine monophosphate–activated protein kinase (AMPK) signaling^6^,^11^. Among these signaling pathways, p38 MAPK may be the most crucial to the mediation of NO toxicity. Factors that can block p38 activation are assumed to protect against NO-induced apoptosis in neural cells^11^,^12^.

Mild hypothermia (32 °C–33 °C) is a well-established therapeutic tool that can be used to alleviate neural injury from various disorders, including hypoxic-ischemic brain damage in newborn infants and acute brain injuries^13^,^14^,^15^,^16^. Studies on the neuroprotective mechanism of mild hypothermia reveal that cold-inducible RNA-binding motif protein 3 (RBM3) plays a crucial role. RBM3 is a glycine-rich protein (17 kDa), and can promote global protein synthesis at both 37 °C and 32 °C by accelerating ribosome assembly, stabilizing mRNA, or decreasing microRNA (miR) expression^17^,^18^,^19^,^20^. In addition to its effect on protein synthesis, RBM3 also plays an important role in cell survival. RBM3 prevents apoptosis caused by hexanedione, staurosporine, contact inhibition, and serum deprivation in neuroblastoma cells and primary neurons^21^,^22^,^23^,^24^. In mouse models of Alzheimer’s disease, RBM3 mediates protective effects of cooling by reducing the loss of synapses^25^,^26^. However, the mechanisms underlying RBM3-conferred neuroprotective effect are not fully understood. Moreover, whether RBM3 or mild hypothermia provides protection against NO-induced neural cell apoptosis has not yet been defined.

The present study showed that both mild hypothermia and RBM3 rescue human SH-SY5Y neuroblastoma cells from NO-induced apoptosis. More importantly, it showed that RBM3 exerts its neuroprotective effects by inhibiting pro-apoptotic p38 signaling pathway. Lastly, miR-143 was found to be a new pro-apoptotic effector, which mediates NO-induced apoptosis in a p38-dependent manner. These data provide new insight into the role of RBM3 in neuroprotection, and the interplay between mild hypothermia, RBM3, p38 signaling, and thermomiR (miR-143).

## Results

### Mild hypothermia (32 °C) protects SH-SY5Y neuroblastoma cells from NO-induced apoptosis

**Figure 1:**
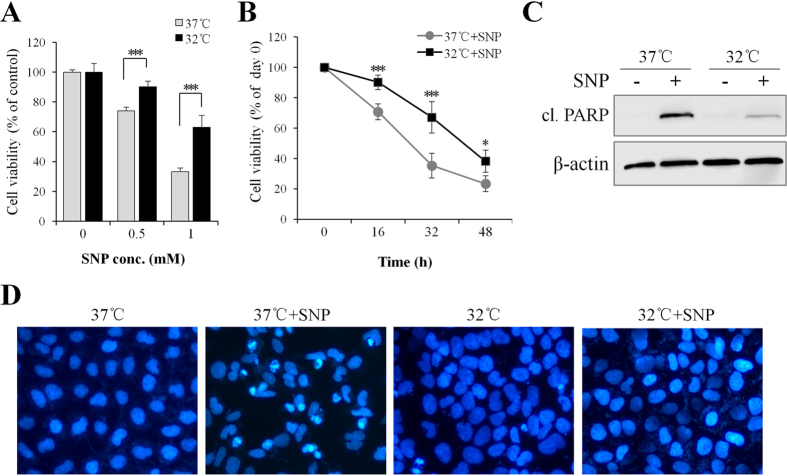
Mild hypothermia (32 °C) prevents SH-SY5Y neuroblastoma cells from NO-induced apoptosis. SH-SY5Y cells were pre-cultured under normothermic (37 °C) or mild hypothermic (32 °C) conditions for 1 d and treated with (**A**) various concentrations of SNP at 37 °C for 16 h, or (**B**) treated with 0.5 mM SNP for 16–48 h, and cell viability was assessed by MTT assay. (**C**) 16 h post SNP (1 mM) treatment, cleaved (cl.) PARP was detected by Western blotting. β-actin served as a loading control. (**D**) 16 h post SNP (1 mM) treatment, cells were stained with DAPI (blue) to evaluate the effects of mild hypothermia on NO-induced apoptosis. *P < 0.05 and ***P < 0.001 versus control group. All results shown are representative of three independent experiments.

The NO donor SNP is a well-established toxin that can trigger apoptosis in cultured neurons and neuroblstoma cells^6^,^12^,^27^,^28^,^29^,^30^,^31^,^32^,^33^. To determine whether cooling protects neural cells from NO-induced apoptosis, human SH-SY5Y neuroblastoma cells were treated with various concentrations of SNP. Cells were pre-cultured at 37 °C (normothermia) or 32 °C (mild hypothermia) for 1 d, and then treated with SNP at 37 °C for 16 h prior to MTT assay. As shown in Fig. 1A and B, SNP induced a dose- and time-dependent cytotoxicity in SH-SY5Y cells, irrespective of temperature profiles (37 °C/37 °C or 32 °C/37 °C) employed. However, when compared to normothermia, mild hypothermia pretreatment significantly increased cell survival, independent of the used SNP concentration (Fig. 1A) or exposure time (Fig. 1B).

The anti-apoptotic effect of mild hypothermia (32 °C) was also assessed by detecting the level of the apoptotic hallmark, caspase substrate poly (ADP-ribose) polymerase (PARP) using Western blotting. As shown in Fig. 1C, hypothermia pretreatment drastically decreased the level of cleaved PARP compared with normothermia. Consistently, DAPI staining showed that hypothermia pretreatment markedly reduced the number of NO-induced apoptotic nuclei in SH-SY5Y cells (Fig. 1D).

Considering that SNP is an NO donor and causes fast, large-scale release of NO, a controlled release NO donor, diethylamine (DEA) NONOate, was also used here to test the anti-apoptotic effect of mild hypothermia (32 °C) on NO insults. Like SNP, DEA NONOate induced a dose- and time-dependent cytotoxicity in SH-SY5Y cells, (Figs S1 and S2). However, mild hypothermia pretreatment significantly protected cells from DEA NONOate-induced cytotoxicity when compared to normothermia (Figs S2 and S3). Notably, hypothermia conferred a more potent protective effect against DEA NONOate than SNP (Figs 1B and S2). Taken together, the preliminary data suggest that mild hypothermia protects SH-SY5Y neuroblastoma cells from NO-induced apoptosis.

### RBM3 silencing mediated by siRNA impedes the neuroprotective effect of mild hypothermia

**Figure 2:**
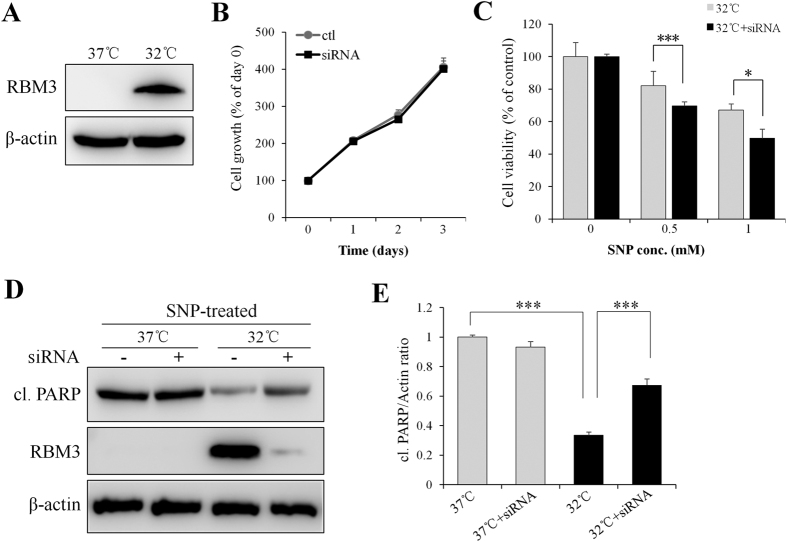
RBM3 silencing by siRNA abolishes neuroprotective effect of mild hypothermia (32 °C). (**A**) SH-SY5Y cells were pre-cultured at 37 °C or 32 °C for 1 d and the induction of RBM3 protein was detected by Western blotting. (**B**) 2 d post transfection of RBM3 siRNA or scramble siRNA (ctl), cells were continued to culture at 37 °C for 1–3 d, and MTT assay was performed to evaluate cytotoxicity of siRNAs. (**C**–**E**) 2 d post transfection of siRNAs, cells were pre-cultured for 1 d at 37 °C or 32 °C and then treated with SNP (1 mM) for 16 h. (**C**) Cell viability was assessed by MTT assay. (**D**) Cleaved PARP was detected by Western blotting. β-actin served as a loading control. (**E**) Histogram analysis for the relative levels of cleaved PARP in panel D. Results were normalized by β-actin. *P < 0.05 and ***P < 0.001 versus control group.

To silence RBM3, the protein level of RBM3 in SH-SY5Y cells cultured at 37 °C and 32 °C was examined. As shown in Fig. 2A, the basal level of RBM3 under normothermic condition (37 °C) was nearly undetectable, while 24 h of hypothermic treatment (32 °C) strongly induced RBM3 expression. Therefore, the silencing effect of RBM3 siRNA was only evaluated in cells pre-cultured at 32 °C. As shown in Fig. 2D, RBM3-specific siRNA decreased the expression of RBM3 protein by more than 90% relative to scrambled siRNA (control). When RBM3 siRNA or scrambled siRNA was transfected into SH-SY5Y cells for up to 3 d, no significant difference in cell proliferation was observed, under either normothermic (Fig. 2B) or mild hypothermic conditions (Fig. S4). However, an MTT assay showed that the neuroprotective effect of mild hypothermia was almost completely abolished when RBM3 was silenced (Fig. 2C). Consistently, Western blot analysis showed that RBM3 silencing substantially abrogated the protective effect of mild hypothermia (Fig. 2D and E). These results indicate that RBM3 plays a crucial role in protecting neural cells from NO-induced apoptosis.

### Overexpression of RBM3 mimics neuroprotective effect of mild hypothermia

**Figure 3:**
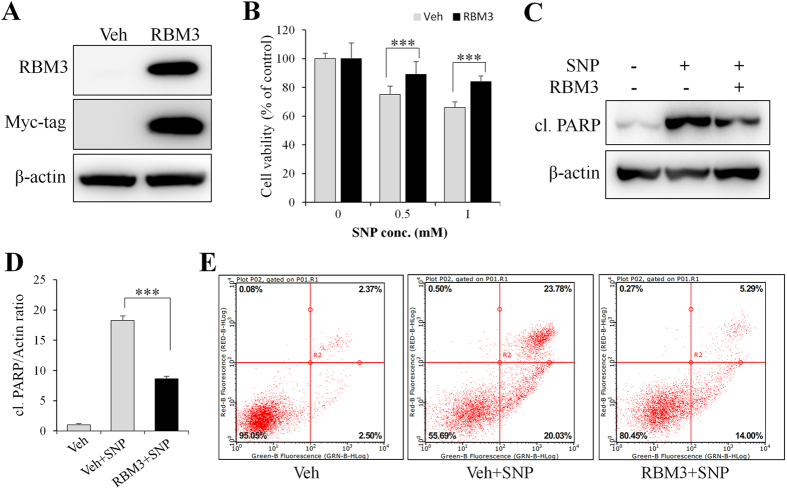
RBM3 overexpression prevents SH-SY5Y cells from NO-induced apoptosis. (**A**) SH-SY5Y cells were transfected with plasmid pXJ40-myc (Veh) or pXJ40-myc-RBM3 (RBM3) for 2 d, and Western blotting was performed to confirm the overexpression of RBM3. β-actin served as a loading control. (**B–E**) 2 d post plasmid transfection, cells were insulted with 1 mM SNP for 16 h. (**B**) Cell viability was assessed by MTT assay, and (**C**) cleaved PARP were detected by Western blotting. (**D**) Histogram analysis for the relative levels of cleaved PARP in panel C. Results were normalized by β-actin. (**E**) Fluorescence-activated cell sorting (FACS) analysis was conducted to evaluate the effect of RBM3 overexpression on NO-induced apoptosis. ***P < 0.001 versus control group.

Next, RBM3 was overexpressed to further confirm its effect on NO-induced apoptosis in SH-SY5Y cells. As shown in Fig. 3A, myc-tagged RBM3 was highly expressed when plasmid pXJ40-myc-RBM3 was transfected into cells. In RBM3-overexpressing cells, cell survival against 0.5 mM SNP increased from 75% to 89% (*P* < 0.001), as shown by the MTT assay (Fig. 3B). Likewise, cell survival against 1 mM SNP increased from 66% to 84% (*P* < 0.001). In line with this, cleaved PARP was also significantly reduced in RBM3-overexpressing cells (Fig. 3C and D) compared with vehicle-transfected control cells.

To consolidate the anti-apoptotic effect of RBM3, annexin V-FITC/PI staining, followed by fluorescence-activated cell sorting (FACS) analysis was performed. As shown in Fig. 3E, the relative number of normal cells in SNP-treated groups was increased from 56% to 80% (*P* < 0.001) when RBM3 was overexpressed. The relative number of late apoptotic cells (annexin V-FITC^+^/PI^+^) was much lower in RBM3-overexpressing cells, further suggesting that RBM3 could mimic the protective effects of mild hypothermia on NO-induced cytotoxicity.

### RBM3 potently inhibits NO-induced p38 activation

**Figure 4:**
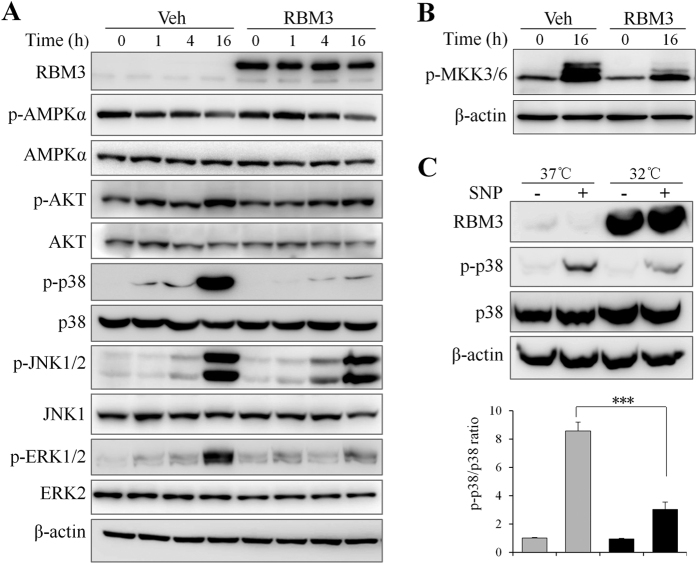
RBM3 potently inhibits NO-induced p38 activation. (**A**) SH-SY5Y cells transfected with plasmid pXJ40-myc (Veh) or pXJ40-myc-RBM3 (RBM3) were treated with SNP (1 mM) for 1–16 h, and Western blotting were performed to detect total and phosphorylated AMPKα, AKT, p38α, JNK1/2, and ERK1/2. **(B**) Western blotting was used to detect the phosphorylated MKK3/MKK6 at 16 h post SNP treatment. (**C**) Cells were pre-cultured at 37 °C or 32 °C for 1 d and treated with SNP for 16 h. Western blotting were performed to detect RBM3 and phosphorylated p38 (p-p38). (**D**) Histogram analysis for the relative levels of p-p38 in panel C. Results were normalized by total p38. ***P < 0.001 versus control group.

As stated above, RBM3 mimicked the protective effects of mild hypothermia, but the underlying mechanism is unclear. Signaling pathways such as AMPK, AKT, and MAPKs p38, JNK, and ERK, have been shown to be involved in NO-induced apoptosis in other cell types^6^,^7^,^8^,^9^,^10^,^11^. Thus, the effects of RBM3 on these signaling pathways were examined. As shown in Fig. 4A, SNP treatment (16 h) induced the activation of AKT, p38, JNK, and ERK, but reduced the activity of AMPK. When RBM3 was overexpressed, SNP-induced activation of AKT, p38, JNK, and ERK at 16 h was reduced more or less, but the reduction of AMPK activity remained unchanged. In particular, RBM3 overexpression almost completely abrogated NO-induced p38 activation, which is known as a stress-related signaling pathway and key mediator of NO cytotoxicity.

To validate the effect of RBM3 overexpression on p38 inhibition, the activation of kinases upstream of p38, mitogen-activated protein kinase kinase 3/6 (MKK3/6), was investigated further. As expected, NO induced a potent activation of MKK3/6, while RBM3 overexpression substantially blocked the activation (Fig. 4B). The next question is whether mild hypothermia also inhibits NO-induced p38 activation. The answer was positive as demonstrated by mild hypothermia significantly decreasing NO-induced p38 activation, when compared to cells cultured at 37 °C (Fig. 4C and D).

The p38 kinase is a well-known MAPK that is activated by stress stimuli, such as cytokines, ultraviolet irradiation, heat shock, and osmotic shock^34^,^35^,^36^, among which transforming growth factor beta 1 (TGF-β1) is a typical and potent inducer. To determine whether RBM3 acts broadly on p38 signaling, the effect of RBM3 overexpression on TGF-β1-induced p38 activation was examined. As shown in Fig. S5, TGF-β1 strongly induced p38 activation, while the overexpression of RBM3 almost completely blocked it, suggesting that RBM3 may broadly regulate p38 signaling.

### p38 signaling is required for NO-induced cytotoxicity

**Figure 5:**
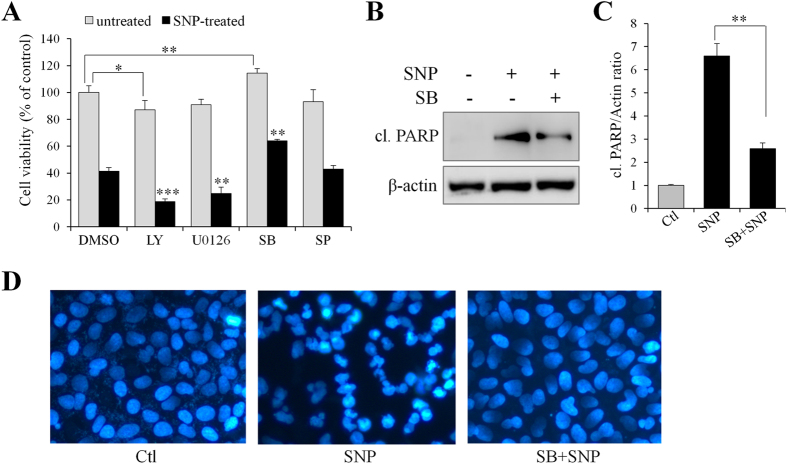
p38 mediates NO-induced apoptosis in SH-SY5Y cells. (**A**) In the presence of various inhibitors, SH-SY5Y cells were treated with SNP (1 mM) for 16 h, and cell viability was assessed by MTT assay. Inhibitors employed includes AKT inhibitor LY294002 (LY, 10 μM), MEK inhibitor U0126 (10 μM), p38 inhibitor SB203580 (SB, 10 μM), and JNK inhibitor SP600125 (SP, 25 μM). (**B**) In the presence of SB203580 (10 μM), cells were treated with SNP for 16 h and cleaved PARP was detected by Western blotting. (**C**) Histogram analysis for the relative levels of cleaved PARP in panel B. (**D**) 16 h post SNP treatment, cells were stained with DAPI (blue) to evaluate the effect of p38 inhibitor (SB) on NO-induced apoptosis. *P < 0.05, **P < 0.01 and ***P < 0.001 versus control group.

The aforementioned results indicate that RBM3 suppresses NO-induced activation of AKT, p38, JNK, and ERK, but whether these signaling pathways contribute to the cytotoxicity of NO is unclear. For this reason, inhibitors specific to these signaling pathways were used. As shown in Fig. 5A, only p38 inhibitor (SB203580) significantly attenuated NO-induced cytotoxicity, whereas AKT inhibitor (LY294002) and MEK inhibitor (U0126) enhanced NO-induced cytotoxicity. In comparison, JNK inhibitor (SP600125) did not significantly affect NO-induced cytotoxicity (Fig. 5A). Next, whether p38 signaling mediates NO-induced apoptosis was further demonstrated. As expected, analysis of the apoptotic marker by Western blotting (Fig. 5B and C) and on apoptotic nuclei by DAPI staining (Fig. 5D) showed that p38 inhibition dramatically rescues cells from NO-induced apoptosis in SH-SY5Y cells. These results are consistent with the observations in other reports regarding to the role of p38 in NO-induced apoptosis in neural cells^11^,^12^.

### RBM3 downregulates NO-induced miR-143 in a p38-dependent manner

**Figure 6:**
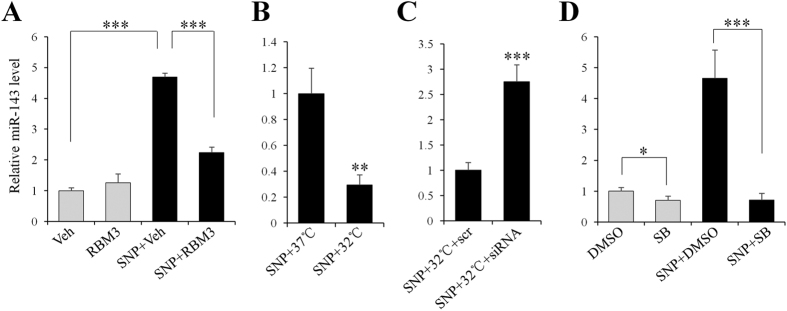
RBM3 downregulates NO-induced miR-143 in p38-dependent way. qPCR was performed to detect miR-143 expression for all the following samples. (**A**) SH-SY5Y cells were transfected with plasmid pXJ40-myc (Veh) or pXJ40-myc-RBM3 (RBM3) for 2 d, and treated with SNP (1 mM) for 16 h. (**B)** Cells were pre-cultured at 37 °C or 32 °C for 1 d and treated with SNP at 37 °C for 16 h. (**C**) 2 d post transfection of RBM3 siRNA or scramble siRNA (scr), cells were treated as shown in panel B. (**D**) In the presence of p38 inhibitor (SB, 10 μM), cells were treated with SNP for 16 h. Gray columns indicate cells without SNP treatment, and black ones indicate cells with SNP treatment. **P < 0.01 and ***P < 0.001 versus control group.

Recently, temperature-sensitive miR-143 was considered a pro-apoptotic factor in some cell models^37^,^38^. Interestingly, one recent report also demonstrated that miR-143 is an RBM3-regulated miRNA, and that it plays a crucial role in modulating the expression of immune-related genes during fever^39^. In this way, it was highly worthwhile to determine whether RBM3 confers protection against NO via regulation on miR-143 in this scenario. As expected, miR-143 was strongly induced in response to SNP stimulation (Fig. 6A), whereas RBM3 overexpression significantly attenuated its induction, indicating that RBM3 downregulates NO-induced miR-143.

To further confirm the inhibitory effects of RBM3/hypothermia on NO-induced miR-143, RBM3 was silenced using siRNA under mildly hypothermic conditions. As expected, mild hypothermia pretreatment blocked much of the induction of miR-143 attributable to NO (Fig. 6B). However, the blockage by hypothermia was substantially abolished when RBM3 was silenced (Fig. 6C).

Most recently, miR-143 was also identified as a downstream target of p38 in muscle cells^40^, but whether it is regulated by p38 in the present scenario is unknown. To test the possibility, p38 inhibitor SB203580 was used. The qPCR results showed that upon p38 signaling inhibition, miR-143 induction by NO was markedly inhibited (Fig. 6D). Taken together, these data indicate that RBM3 downregulates NO-induced miR-143 by interfering with p38 signaling.

### miR-143 mediates NO-induced apoptosis

**Figure 7:**
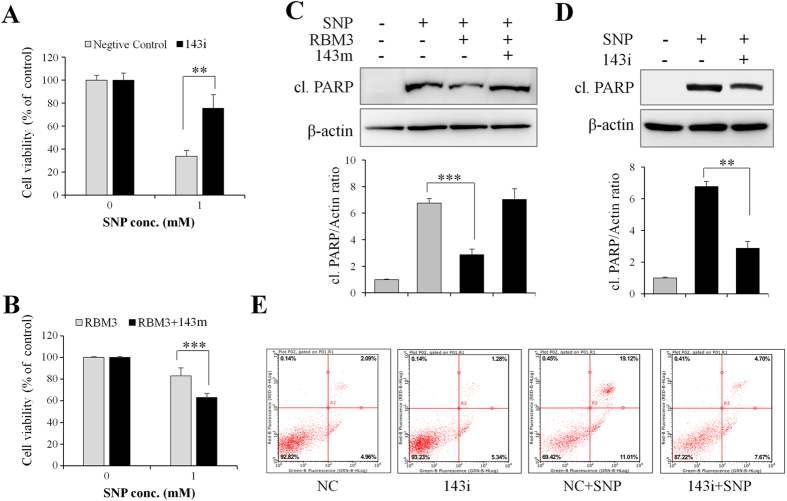
miR-143 mediates NO-induced apoptosis. SH-SY5Y cells were transfected with miR-143 inhibitor (143i) or negative control (NC) inhibitor for 2 d, and treated with SNP (1 mM) for 16 h. (**A**) Cell viability was assessed by MTT assay, (**D**) cleaved PARP was detected with Western blotting, and (**E**) FACS analysis was conducted to evaluate the effect of miR-143 inhibitor on NO-induced apoptosis. (**B** and **C**) RBM3-overexpressing cells were transfected with miR-143 mimics (RBM + 143 m) or NC mimics for 2 d, and then treated with SNP for 16 h. (**B**) Cell viability was assessed by MTT assay, and (**C**) cleaved PARP was detected with Western blotting. The lower panels in C and D are histogram analysis for corresponding levels of cleaved PARP. *P < 0.05, **P < 0.01 and ***P < 0.001 versus control group.

Lastly, it was determined whether the downregulation of miR-143 by RBM3 confers a protective effect on NO-induced apoptosis. As shown in Fig. 7A, transfection of miR-143 inhibitor oligonucleotide (143i) into the cells significantly reduced the cytotoxicity of NO. Instead, transfection of miR-143 mimics oligonucleotide (143 m) into RBM3-overexpressing cells markedly attenuated RBM3-conferred cell protection against NO (Fig. 7B).

As stated above, RBM3 overexpression significantly reduced the level of cleaved PARP induced by NO (Fig. 7C). However, the addition of miR-143 mimics (143 m) substantially reversed it (Fig. 7C). In line with this evidence, when the activity of endogenous miR-143 was blocked by miR-143 inhibitor (143i), NO-induced PARP cleavage was markedly reduced (Fig. 7D).

To further confirm the role of miR-143 in NO-induced apoptosis, annexin V-FITC/PI staining, followed by FACS analysis was performed. Compared to negative control (NC) inhibitor, miR-143 inhibitor (143i) significantly reduced NO-induced apoptosis (Fig. 7E). In conclusion, these data suggested that RBM3 prevents cells from NO-induced apoptosis by inhibiting the expression of pro-apoptotic miR-143.

## Discussion

The present study demonstrated that mild hypothermia protects neuroblastoma cells from NO-induced apoptosis and that this protection is mediated by the cold-inducible protein RBM3. Results also showed that RBM3 exerts a neuroprotective effect against NO insults mainly through blocking p38 MAPK signaling. Furthermore, a thermomiR, miR-143, was identified as a new pro-apoptotic effector of NO cytotoxicity. These findings may represent a new molecular mechanism responsible for RBM3-conferred neuroprotection.

Hypothermia is an effective therapeutic tool used to reduce neurologic impairment. In the 1950 s, hypothermia was used to alleviate the side effects of brain surgery^41^. Even today, cooling an infant’s body to 33 °C is still the only available therapy for neonatal asphyxia^22^. The present study provided convincing evidence that hypothermia prevents NO-induced cytotoxicity in human neuroblastoma cells via inducing RBM3. First, accompanied by elevated levels of RBM3 protein, hypothermia protected cells from NO-induced apoptosis. Second, RBM3 silencing using a siRNA approach abolished the hypothermia-conferred neuroprotective effect. Third, the overexpression of RBM3 mimicked hypothermia-conferred neuroprotection against NO-induced apoptosis. As reported previously, RBM3 upregulation in response to hypothermia is required for hypothermia-induced neuroprotection against various insults. Because cooling the human brain to 32 °C–33 °C is impracticable and that can even lead to serious injuries to the brain. Inducing RBM3 specifically in injured brain areas may be a promising therapy for neurodegenerative diseases. Unfortunately, this issue has not yet been fully explored.

Stress-activated signaling pathways, including p38 and JNK, appear to be critical to induce apoptosis in neurons. Previous studies of NO-induced apoptosis demonstrated that both p38 and JNK are complicated in NO toxicity^11^,^12^,^42^. However, JNK may contribute less to the NO toxicity, than p38 activation does. The present study demonstrated a strong activation of p38 and JNK at 16 h post NO insults, but the following assays with inhibitors showed that p38, is critically required for NO toxicity, but JNK is not. These results are consistent with the findings of previous studies, supporting the conclusion that p38 signaling mainly mediates NO-induced apoptosis in neural cells.

The most important finding of the present study is that RBM3 executes its neuroprotective effect via inhibition of NO-induced p38 signaling in neuroblastoma cells. More convincingly, RBM3 also inhibits TGF-β1-induced p38 activation in SH-SY5Y cells, suggesting that RBM3 may broadly regulate p38 signaling. This is the first evidence that cold-inducible protein RBM3 directly interferes with p38 signaling. p38 signaling is associated with apoptosis in numerous types of cells and activated by various cellular stresses, such as x-ray/ultraviolet irradiation, heat/osmotic shock, and oxidative/nitrosative stress and pro-inflammatory cytokines such as TGF-β1^43^. In this way, inhibition of p38-mediated apoptosis in neurons mitigates neurodegenerative disease. However, it is still unknown how RBM inhibits NO-induced p38 activation. It is known, however, that NO donors (such as SNP and DEA NONOate) activate p38 signaling via induction of reactive oxygen species (ROS)^44^,^45^,^46^,^47^, so it is worthwhile to investigate whether RBM3 inhibits p38 signaling by reducing NO-elicited ROS.

One important role of RBM3 is to accelerate global protein synthesis via regulating miRNAs in cells^20^. One previous report showed that RBM3 could directly bind to precursor miRNAs and overcome the intrinsic inhibitory influence of pre-miRNA ribonucleoprotein complex processing at the Dicer step^18^. In a more recent study, Wong *et al*. demonstrated that a reduced expression of RBM3 by fever results in an increased expression of temperature-sensitive miRNAs (thermomiRs), including miR-143^39^. For these reasons, we determined whether RBM3 is involved in the regulation of miR-143 in a direct-binding way. Using co-immunoprecipitation (co-IP) and anti-myc tag beads, myc-tagged RBM3 was isolated from RBM3-overexpressing cells and PCR for pre-miR-143 was performed. Unexpectedly, no pre-miR-143 product could be detected from IP products of myc-tagged RBM3 (data not shown), thus ruling out the possibility that RBM3 regulates miR-143 in a direct way. In combination, these data suggest that RBM3 might not affect the biogenesis of miR-143 directly, but it may inhibit the induction of miR-143 indirectly, namely by interfering with p38 signaling.

The second important finding of this study is that miR-143 acts downstream of p38 signaling and mediates NO-induced apoptosis. The conclusion was supported by three facts. First, miR-143 was strongly induced on NO stimuli, whereas p38 inhibitor diminished the induction. Second, miR-143 inhibitor attenuated NO-induced apoptosis. Third, miR-143 mimics abolished RBM3-conferred protective effect. In most studies, miR-143 was found to be highly expressed in normal tissues and downregulated in many cancers, indicating that miR-143 might function as a potential tumor suppressor^48^,^49^. In a recent report, the researchers demonstrated that miR-143 could inhibit the expression of Bcl-2 and cause caspase-3 activation, thus inducing apoptosis in osteosarcoma cells^38^. However, no obvious alteration of Bcl-2 was observed at the protein level, under conditions of either hypothermia or RBM3 overexpression (data not shown). For this reason, more mechanisms regarding to pro-apoptotic effect of miR-143, should be explored in the future.

Since SNP was frequently used as an apoptosis inducer to investigate the anti-apoptotic effects of various drugs in previous reports, SNP was chosen to treat neuroblastoma cells SH-SY5Y in this work. However, it is necessary to point out that the interaction between NO and neuroblastoma cells is different from the interaction between NO and normal neuronal cells. Neuroblastoma cells generate extracellular superoxide anions through NADPH oxidase 1 (NOX1) and the rapid interaction between superoxide anions and NO yields peroxynitrite that can form peroxynitrous acid and generate apoptosis-inducing hydroxyl radicals^46^,^47^. As neuroblastoma cells, in contrast to nonmalignant cells express membrane-associated catalase^46^, exogenous NO derived from NO donors may be oxidated and eventually generated peroxynitrite will be decomposed. Therefore, it can be predicted that NO-dependent effects on these tumor cells will require higher concentrations of NO than nonmalignant cells. For this reason, a controlled release NO donor, DEA NONOate, was also used to confirm the hypothermia-conferred protective effect on NO-induced apoptosis. When DEA NONOate was used as an NO donor, cells underwent apoptotic patterns similar to those associated with SNP. Somewhat differently, hypothermia prevented DEA NONOate-induced apoptosis to a greater extent than SNP.

In summary, the present study indicates that the cold-inducible protein RBM3 prevents NO-induced apoptosis in human neuroblastoma cells by modulating p38 signaling and miR-143. Hypothermia and RBM3 prevent apoptosis in neuroblastoma cells, indicating that people could utilize RBM3 induction or RBM3 agonist to improve neurological disorders in injured brain areas. p38 mediates NO toxicity through upregulating pro-apoptotic miR-143, revealing that miR-143 might be considered as a potential target in improving neurodegenerative diseases caused by NO toxicity. Finally, results demonstrated that RBM3 might regulate p38 MAPK signaling in a broad range, suggesting that RBM3 might be used for the therapy of p38-related diseases, such as fibrosis.

## Materials and Methods

### Materials

SNP and diethylamine (DEA) NONOate were purchased from Sigma Aldrich (MO, USA). The RBM3 antibody (ab134946) was from Abcam (Cambridge, MA, USA), and antibodies against, AMPKα (#5832), p-AMPKα (#2535), AKT (#9272), p-AKT (#4060), p-ERK1/2 (#4370), p38 (#9212), p-p38 (#4511), p-JNK1/2 (#4668), p-MKK3/MKK6 (#12280), cleaved PARP (#9541), and β-actin (#4970) were form Cell Signaling Technology (Beverly, MA, USA). Antibodies against ERK1 (sc-93), and JNK1 (sc-474) were from Santa Cruz Biotechnology (CA, USA). The 4,6-diamidino-2-phenylindole dihydrochloride (DAPI) was purchased from Sangon (Shanghai, China). Annexin V-FITC cell apoptosis kit was from Beyotime (Jiangsu, China). Inhibitors SB203580 (p38 MAPK) and SP600125 (JNK), were from Sigma Aldrich. Inhibitors LY294002 (AKT) and U0126 (MEK) were from Cell Signaling Technology. The siRNAs specific to human *Rbm3*, human miR-143 mimics and antisense inhibitor, and their negative control oligonucleotides were purchased from GenePharma (Shanghai, China).

### Cell culture and drug treatment

Human neuroblastoma cell line SH-SY5Y cells (gifted by Dr. Evelyne Goillot, Laboratoire d’Immunologie, Centre Leon Berard, France and Eva Feldman, University of Michigan, MI, USA) were maintained in Dulbecco’s modified Eagle’s medium (DMEM) supplemented with 10% fetal calf serum (FCS), 100 U/mL penicillin, and 100 μg/mL streptomycin in a humidified atmosphere containing 5% CO_2_ in air at 37 °C. For mild hypothermia experiments, cells were pre-cultured for 1 d at 32 °C and then transferred to 37 °C further culture or treatment.

SNP or DEA NONOate (dissolved in ethanol) at various final concentrations was added to the cells for a time course up to 48 h as indicated. In most cases, SH-SY5Y cells were treated with 1 mM SNP for 16 h at 37 °C. The SB203580 (10 μΜ), SP600125 (25 μΜ), LY294002 (10 μΜ), and U0126 (10 μΜ) were used as inhibitors specific to p38, JNK, AKT, and ERK. The cells were incubated with inhibitors for 1 h prior to SNP or DEA NONOate treatment.

### Overexpression of RBM3

The sequence of the human *Rbm3* gene (GenBank NM_006743.4) was resynthesized chemically by Sangon (Shanghai, China) and cloned into the expression vector pXJ40-myc. The RBM3-containing vector pXJ40-myc-RBM3 was transfected into SH-SY5Y cells using Lipofectamine 2000 (Invitrogen, CA, USA), and the SH-SY5Y cells transfected with vehicle pXJ40-myc served as a control. Generally, 2 d post transfection, cells were used for further experiments. All transfections were performed according to the manufacturer’s instructions.

### Short interfering RNA

To knockdown human RBM3 mRNA (GenBank NM_006743.4), an RBM3-specific short interfering RNA (siRNA) target the coding region was designed. The specific siRNA (5′-CCA UGA ACG GAG AGU CUC UTT-3′) and a scrambled siRNA (5′-UUC UCC GAA CGU GUC ACG UTT-3′) not matching with any of the human genes were synthesized by GenePharma (Shanghai, China). When cells in six-well plates reached to 70–80% confluence, cells were transfected with siRNA (10 nM) using Lipofectamine 2000. At 48 h post transfection, cells were used for further experiments.

### MiRNA transfection

Growing SH-SY5Y cells were transfected with 20 nM miR-143 mimics (5′-UGA GAU GAA GCA CUG UAG CUC-3′) or the negative control (NC) mimics (5′-UUC UCC GAA CGU GUC ACG UTT-3′), antisense miRNA inhibitor (5′-GAG CUA CAG UGC UUC AUC UCA-3′) or the NC inhibitor (5′-CAG UAC UUU UGU GUA GUA CAA-3′) using Lipofectamine 2000, following the manufacturer’s instruction. RNA or protein was harvested 48 h after transfection, and miRNA expression level was quantified with qPCR.

### RNA extraction and real-time PCR (qPCR)

Total RNA was extracted from SH-SY5Y using TRIzol (Thermo Fisher Scientific Inc, USA) following the manufacturer’s instruction, and cDNA synthesis was carried out with a first strand cDNA synthesis kit (Thermo Fisher Scientific Inc, USA). Primers were synthesized by Genewiz Biotechnology (Suzhou, China). The following primers were used: miR-143: Primer-F: 5′-ACA CTC CAG CTG GGT GAG ATG AAG CAC TG-3′, U6: Primer-F: 5′- CTC GCT TCG GCA GCA CA -3′, Universal Reverse Primer (URP): 5′-TGG TGT CGT GGA GTC G-3′. We used the 2^−ΔΔCt^ method for normalization of raw data as described^50^.

### Cell viability assay

The viability of cells was assessed using an MTT assay. In brief, 2 × 10^4^ SH-SY5Y cells (200 μL) were seeded in 96-well plates. Following 12-h incubation, the medium was replaced with a fresh one containing SNP or DEA NONOate at various concentrations for up to 48 h as indicated. In most cases, SH-SY5Y cells were treated with 1 mM SNP for 16 h at 37 °C. After that, cells were incubated with 1 mg/mL MTT for 4 h at 37 °C and then the medium was removed. The incubated cells were extracted with dimethyl sulfoxide (Sigma), and the absorbance was measured at a wavelength 490 nm using a SpectraMax Plus absorbance microplate reader (Molecular Devices, USA).

### Western blotting analysis

After treatment, SH-SY5Y cells were washed twice with cold PBS (3.2 mM Na_2_HPO_4_, 0.5 mM KH_2_PO_4_, 1.3 mM KCl, 140 mM NaCl, pH 7.4) and then proteins were extracted with RIPA (Vazyme Biotech, Nanjing, China) and centrifuged at 15,000 × g for 10 min at 4 °C. The protein concentration was measured using a Protein Assay Kit II (BioRad, Hercules, USA). 40 μg of protein samples was resolved on a 8% or 15% SDS-PAGE and transferred onto a polyvinylidene difluoride membrane (Millipore, Billerica, CA, USA), and then blocked with PBS-T (0.1% Tween-20 in PBS) containing 5% non-fat milk. After blocking, the membranes were incubated at 4 °C overnight with different primary antibodies. After washing with PBS for 3 times (5 min each), the membranes were further incubated with horse radish peroxidase-conjugated secondary antibodies (Vazyme Biotech, Nanjing, China) and developed using Pierce’s West Pico Chemiliuminescence substrate. The immunoreactive bands were visualized by a Luminescent image analyzer (Amersham Imager 600, GE Healthcare). The immunoreactive protein band density was measured by the software ImageJ 1.50 (NIH).

### DAPI staining

The SH-SY5Y cells were seeded in 12-well plates. After treatment, the cells were washed once with cold PBS and fixed with 4% paraformaldehyde for 30 min. Next, they were washed with cold PBS three times and then incubated with 5 μg/mL DAPI solution for 10 min. Finally, the cells were washed with cold PBS three times and examined using a fluorescence microscope (Carl Zeiss, NY, USA).

### Annexin-V apoptosis assay

Apoptotic cells were identified by annexin V-FITC cell apoptosis kit (Beyotime Biotechnology, China) following the manufacturer’s instruction. Briefly, SH-SY5Y cells were transfected with pXJ40-myc-RBM3 for 2 d and then treated with 1 mM SNP for 24 h. Thereafter, cells and cultural supernatants were collected by centrifugation at 1,000 rpm for 5 min, washed with cold PBS twice and gently resuspended in 195 μL binding buffer. Then 5 μL annexin V-FITC and 10 μL propidium solution were added and incubated with cells in the dark for 20 min. Apoptosis rates were analyzed by flow cytometry (Guava, Merck Millipore).

### Statistical analysis

Data were expressed as mean ± S.E. values. The group means were compared by analysis of variance, and the significance of differences was determined by *post-hoc* testing using Bonferroni’s method. Differences were considered significant at *P* < 0.05.

## Additional Information

**How to cite this article:** Yang, H.-J. *et al*. RNA-binding protein RBM3 prevents NO-induced apoptosis in human neuroblastoma cells by modulating p38 signaling and miR-143. *Sci. Rep.* **7**, 41738; doi: 10.1038/srep41738 (2017).

**Publisher's note:** Springer Nature remains neutral with regard to jurisdictional claims in published maps and institutional affiliations.

## Supplementary Material

Supplemental Data
