# Supplementary material for: RNA-binding protein RBM3 prevents NO-induced apoptosis in human neuroblastoma cells by modulating p38 signaling and miR-143
Source: Sci Rep. 2017 Jan 30;7:41738. doi: 10.1038/srep41738 (PMC5278414; doi:10.1038/srep41738)
Supplement: Supplemental Data [file srep41738-s1.pdf]

RNA-binding protein RBM3 prevents NO-induced apoptosis in human neuroblastoma cells by modulating p38 signaling and miR-143

Hai-Jie Yang[1,2,3]*, Fei Ju[1,2], Xin-Xin Guo[1,2], Shuang-Ping Ma[1], Lei Wang[1], Bin-Feng Cheng[1], Rui-Juan Zhuang[1], Bin-Bin Zhang[1], Xiang Shi[1], Zhi-Wei Feng[2]*, Mian Wang[1,2]

[1]School of Life Science and Technology, Xinxiang Medical University, Xinxiang 453003, China

[2]Henan Collaborative Innovation Center of Molecular Diagnosis and Laboratory Medicine, Xinxiang Medical University, Xinxiang 453003, China

[3]Henan Key Lab of Biological Psychiatry, Second Affiliated Hospital of Xinxiang Medical University, Xinxiang 453003,China

*Address correspondence to:

Dr. Hai-Jie Yang or Dr. Zhi-Wei Feng

601 Jinsui Road, Xinxiang, Henan, 453003, China.

e-mail: hjyang_wmd@hotmail.com (H. J. Yang)

xxyxy_fzw@163.com (Z. W. Feng)

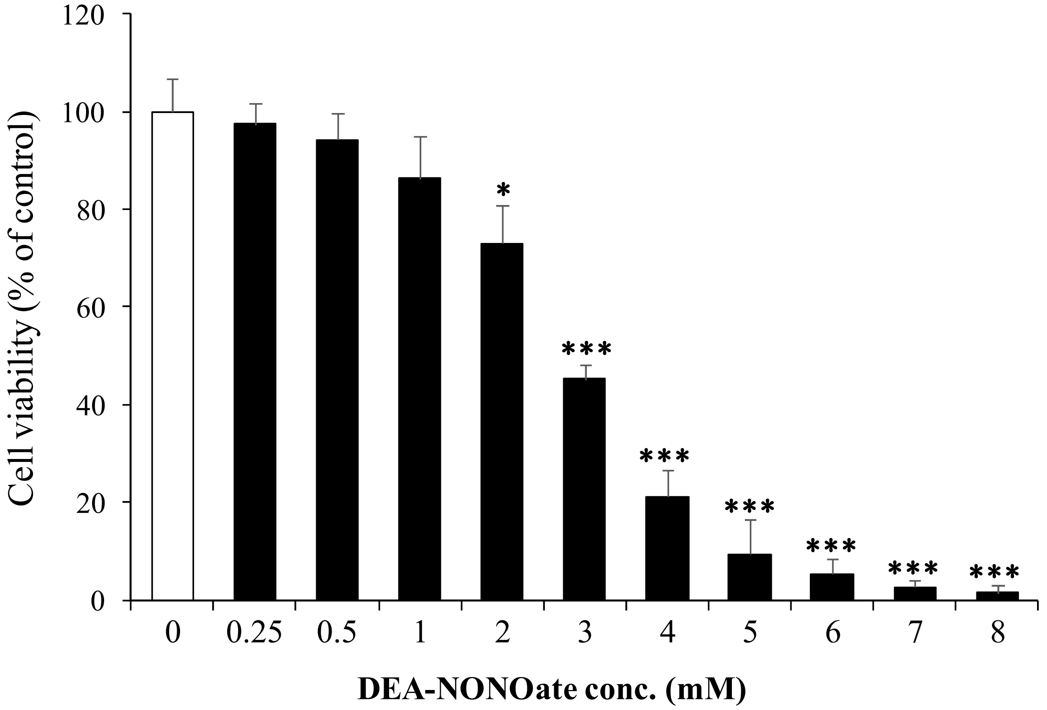

Figure S1. DEA NONOate induces a dose-dependent cytotoxicity in SH-SY5Y neuroblastoma cells. Cells were treated various concentrations of DEA NONOate for 16 h, and cell viability was assessed by MTT assay. $*P < 0.05$ and $***P < 0.001$ versus control group. All results shown are representative of three independent experiments.

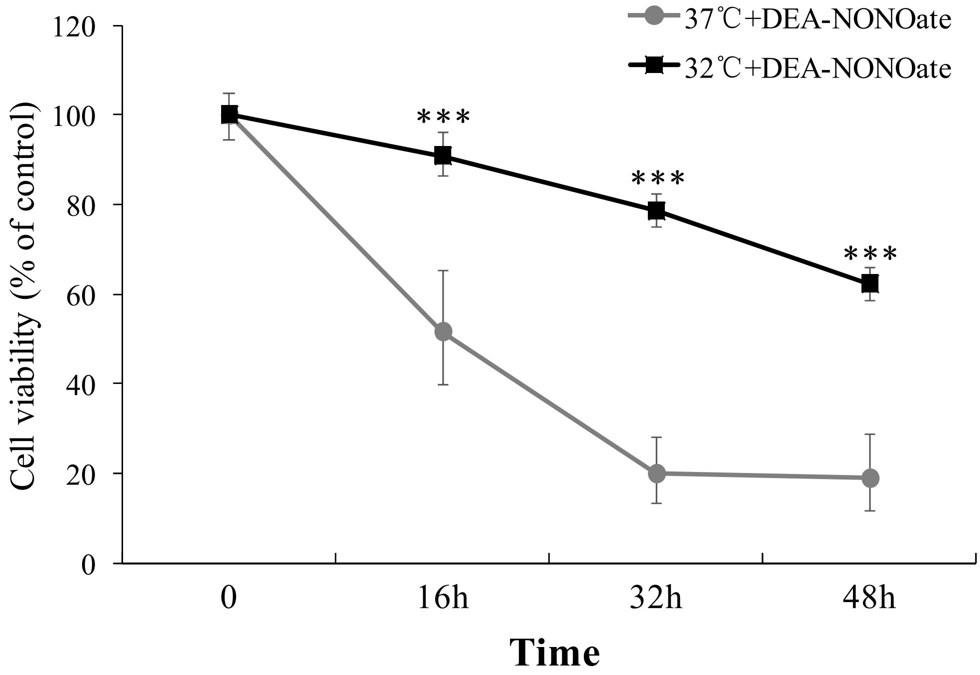

Figure S2. DEA NONOate induces a time-dependent cytotoxicity in SH-SY5Y cells and mild hypothermia (32ºC) reduces its cytotoxicity. SH-SY5Y cells were pre-cultured under normothermic (37ºC) or mild hypothermic (32ºC) conditions for 1 d and treated with DEA NONOate (3 mM) for 16−48 h, and then cell viability was assessed by MTT assay. ***$P < 0.001$ versus control group.

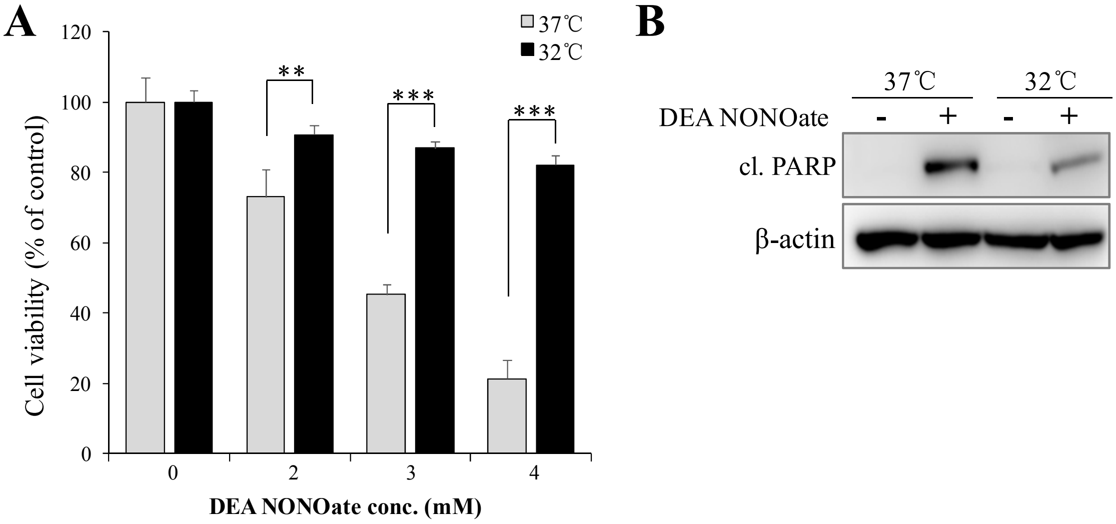

Figure S3. Mild hypothermia prevents SH-SY5Y neuroblastoma cells from DEA NONOate-induced apoptosis. SH-SY5Y cells were pre-cultured under normothermic (37ºC) or mild hypothermic (32ºC) conditions for 1 d and treated with DEA NONOate (3 mM) for 16 h. (**A**) The cell viability was assessed by MTT assay, and (**B**) cleaved (cl.) PARP was detected by Western blotting. β-actin served as a loading control. $^{**}P < 0.01$ and $^{***}P < 0.001$ versus control group.

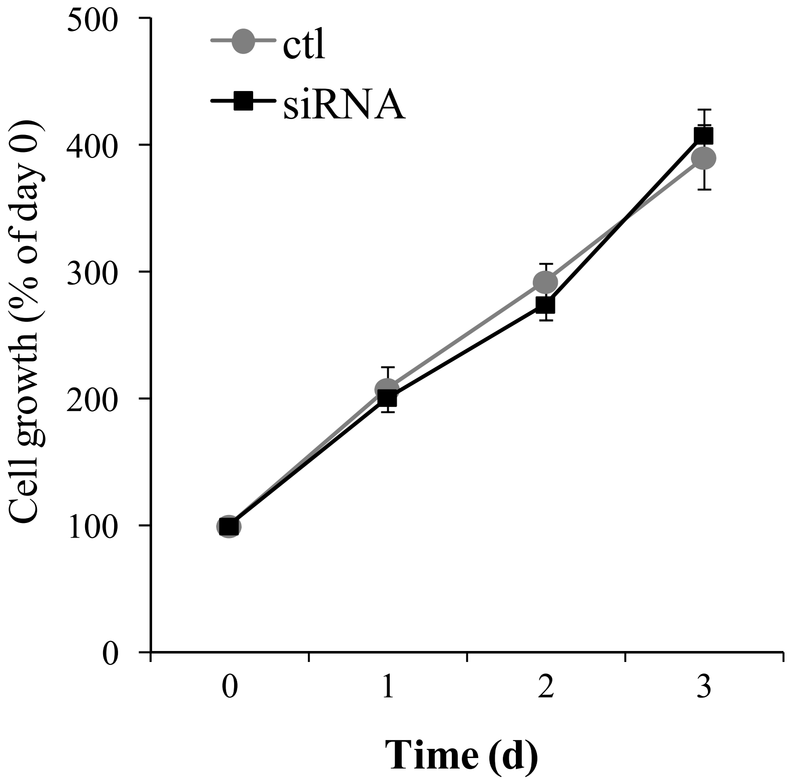

Figure S4. Effect of siRNAs on cell growth of SH-SY5Y under hypothermic conditions. 2 d post transfection of RBM3 siRNA or scramble siRNA (ctl), SH-SY5Y cells were continued to culture at 32ºC for 1−3 d, and MTT assay was performed to evaluate cytotoxicity of siRNAs under hypothermic conditions.

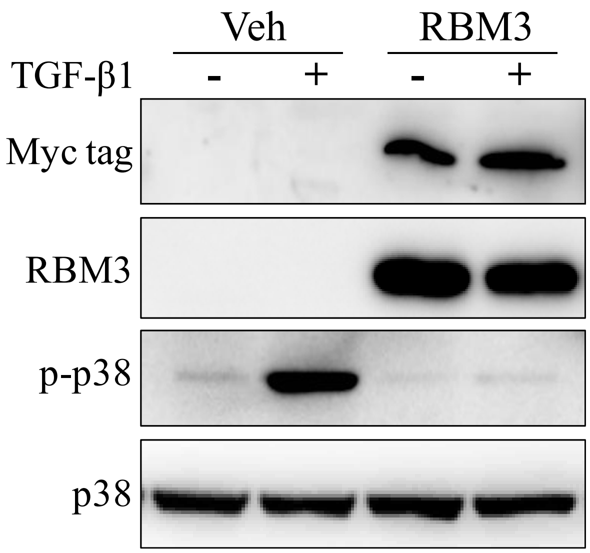

Figure S5. RBM3 inhibits TGF-β1-induced p38 activation. SH-SY5Y cells were transfected with plasmid pXJ40-myc (Veh) or pXJ40-myc-RBM3 (RBM3) for 2 d, and then treated with TGF-β1 (5 ng/mL) for 30 min. Western blotting was performed to detect the levels of phosphorylated p38 (p-p38). The total p38 served as a loading control.
